# Sex-specific formulations of doxazosin mesylate via direct powder extrusion 3D printing

**DOI:** 10.1007/s13346-025-01862-4

**Published:** 2025-04-22

**Authors:** Patricija Januskaite, Alvaro Goyanes, Mine Orlu, Abdul W. Basit

**Affiliations:** 1 Department of Pharmaceutics, UCL School of Pharmacy, University College London, 29-39 Brunswick Square, London, WC1N 1AX UK; 2 FABRX Ltd., Henwood House, Henwood, Ashford, Kent, TN24 8DH UK; 3 FABRX Artificial Intelligence, Calle Enrique Vidal Abascal 7, Santiago de Compostela, 15702 Spain; 4 Departamento de Farmacología, Farmacia y Tecnología Farmacéutica, Facultad de Farmacia, Instituto de Materiales (iMATUS) and Health Research Institute of Santiago de Compostela (IDIS), Universidade de Santiago de Compostela, Santiago de Compostela, 15782 Spain

**Keywords:** Additive manufacturing of oral drug products, Three-dimensional printing of medications, Personalized therapeutics, Modified release formulations and pharmaceuticals, Hypertension, Gender specific drug prescribing

## Abstract

Males and females are known to exhibit significant differences in drug pharmacokinetics and pharmacodynamics, which are still overlooked in pharmaceutical research and development. These disparities contribute to adverse effects and increased mortality in females, highlighting the critical need for sex-specific formulations. Extended-release formulations of doxazosin mesylate, an alpha blocker used to treat hypertension, have shown significant sex-based differences in pharmacokinetics, leading to heightened adverse effects in females and rendering current titration recommendations impractical. This study explored the potential of a 3D printing (3DP) technology, direct powder extrusion (DPE), for producing personalised, sex-specific doses of doxazosin mesylate. A simple three component formulation was made composed of hydroxypropyl cellulose (HPC) polymer Klucel JF, D-mannitol, and doxazosin mesylate. Extended-release printlets of varying doses (1, 2, and 3 mg) were manufactured from a single 1% w/w doxazosin pharma-ink batch, enabling easy dose personalisation by adjusting the printlet dimensions. The use of a single pharma-ink supports the technology’s ease of use in a pharmacy setting, by eliminating frequent pharma-ink changes during the pharmaceutical compounding process. In vitro dissolution testing revealed an extended drug release profile, influenced by surface-area-to-volume (SA: V) ratios. Introducing channels in larger printlets standardized the SA: V ratios, enhancing release profile uniformity. Release kinetics followed the Hixson-Crowell and Korsmeyer-Peppas models, indicating diffusion and polymer swelling mechanisms. This work highlights the capability of DPE 3DP for creating personalized, extended-release oral dosage forms, supporting precise dose customization for patient-specific therapy.

**Supplementary Information:**

The online version contains supplementary material available at 10.1007/s13346-025-01862-4.

**Figure:**
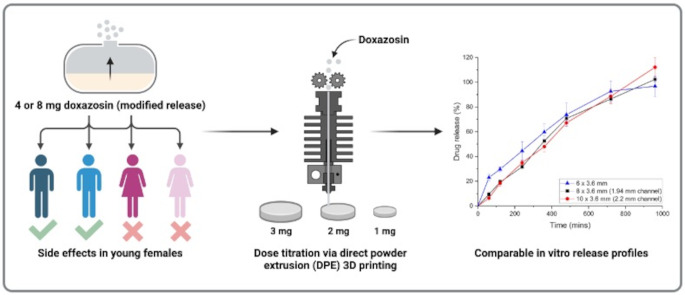
Graphical Abstract

## Introduction

Despite males and females making up roughly equal proportions of the global population, a pronounced sex bias persists in the pharmaceutical space. Historically, preclinical research and drug development studies have predominantly relied on male cells and tissues, male animal models, and male participants [1–4]. Sex-based differences in drug pharmacokinetics and pharmacodynamics are common in many pharmaceutical products today, such as diazepam where females have a higher distribution volume compared to males, which is reversed when taken with alcohol [5]. Other examples include differences in hepatic clearance, with drugs like digoxin, zolpidem, temazepam, and paracetamol having a greater hepatic clearance in males; whilst being greater in females for verapamil, cyclosporine, and erythromycin [2, 6–8]. Failing to account for these differences often leads to serious side effects and increased mortality in females, underscoring the urgent need for more sex-specific formulations [9].

Hypertension, one of the most prevalent chronic conditions, is defined by persistently elevated arterial blood pressure, making it a major risk factor for serious disorders such as heart disease and stroke [10]. Affecting over 1.3 billion adults worldwide [11], it is largely influenced by environmental and lifestyle factors, making it a preventable condition. However, many individuals still require antihypertensive medications despite lifestyle interventions [12]. Doxazosin mesylate, a biopharmaceutical classification system (BCS) class II drug, is an alpha-blocker used to treat hypertension [13, 14]. Approved by the FDA in 2005, doxazosin is available as immediate release (Cardura™) or controlled release (Cardura XL™) tablets, with doses of 4 mg or 8 mg per day. The controlled release formulation uses a gastrointestinal therapeutic system (GITS) for precise plasma control and simplified dosing [15, 16].

A 2005 FDA clinical pharmacology and biopharmaceutics review examined the pharmacokinetics of doxazosin, comparing young and elderly males and females to assess age- and sex- related effects [17]. Findings revealed that the 4 mg extended-release formulation demonstrated a significant sex effect, with 45% higher C_max_ and 46% higher AUC values in young females compared to young males. Additionally, young females reported the highest number of adverse events (34), 48% of which occurred on the first day of treatment. As a result, the FDA recommends dose titration starting at 1 mg/day for young females; however, the lowest available dose for extended-release doxazosin is 4 mg, making titration unfeasible. Furthermore, the GITS cannot be cut or crushed without compromising its controlled release mechanism. To address these limitations, three-dimensional (3D) printing (3DP) offers a promising solution by enabling the creation of personalised doses tailored to specific populations [18–21]. Through a layer-by-layer manufacturing process, 3DP ensures precise dosing while preserving the drug’s extended-release profile, making it particularly valuable for patients like young females who require careful dose adjustments for optimal safety and efficacy.

Since the advent of pharmaceutical 3DP, extrusion-based techniques like fused deposition modelling (FDM) and semi-solid extrusion (SSE) have been widely explored due to their affordability and ease of use [22–30]. However, both techniques have drawbacks, limiting their use in pharmaceutical applications. SSE 3DP requires complete solvent evaporation after printing which requires extensive drying times and may complicate manufacture [31]. Additionally, the gel-like semi-solid material used carries the risk of shape loss or material collapse post-printing [32]. In contrast, FDM 3DP does not exhibit such post-processing issues but is time consuming due to the filament production process of hot melt extrusion (HME), which must meet specific mechanical and rheological properties [33, 34]. Furthermore, the dual use of thermal processes, both in HME and FDM, can lead to drug degradation, making FDM unsuitable for drugs with low melting points or temperature sensitivity [35, 36].

To overcome the limitations associated with extrusion-based techniques, a novel additive manufacturing method, direct powder extrusion (DPE), was introduced. First used in 2019 by Goyanes et al., to print itraconazole and hydroxypropyl cellulose (HPC) loaded tablets (known as Printlets™) [37], this innovative single-step manufacturing process eliminates the need for filament production, utilising powder directly. The versatility and suitability of DPE 3DP for pharmaceutical manufacture under Good Manufacturing Practice (GMP) conditions has already been demonstrated by leading companies such as FABRX Ltd. and Triastek [38, 39]. As a relatively new technology, research into pharmaceutical DPE 3DP is still in its early stages. To date, only a limited number of investigational dosage forms have been explored, including modified-release tablets of various active pharmaceutical ingredients (APIs) [38], immediate-release caffeine [40] and paracetamol tablets [41], paediatric formulations of praziquantel [42], and opioid medicines with abuse and alcohol deterrent properties [43]. The technology has also been used to manufacture minitablets (< 100 mg) with high loadings of nifedipine [44], as well as cyclodextrin based tablets of niclosamide to enhance solubility [45], and paediatric budesonide minitablets [46]. Given its advantages, this innovative technology holds significant potential for printing titrated doses on demand in a pharmacy or hospital setting, in the form of pharmaceutical compounding [47].

This study aims to leverage DPE 3DP to address the critical need for sex-specific dosing, focusing on alleviating side effects associated with antihypertensive treatments like doxazosin mesylate. The work explores the feasibility of producing tailored, extended-release oral tablets, with minimal excipients that may introduce more sex differences, that maintain comparable release profiles while enabling precise dose titration. This approach seeks to overcome the limitations of traditional drug formulations, paving the way for safer and more effective personalized medicines, particularly for females that are disproportionately affected by standard dosing practices.

## Materials and methods

### Materials

Doxazosin mesylate (547.58 g/mol) was purchased from MedChemExpress LLC (NJ, USA). Klucel HPC polymer JF (MW 140,000 g/mol) was purchased from Ashland (DE, USA). D-mannitol (MW 182.17 g/mol) and methanol puriss grade (≥99.8) were purchased from Sigma-Aldrich (Gillingham, UK). Potassium dihydrogen orthophosphate (KH_2_PO_4_) (MW 136.09 g/mol) was secured from Avantor (Leicestershire, UK). Sodium hydroxide (NaOH) 5 M was acquired from Scientific Laboratory Supplies (Nottingham, UK). Hydrochloric acid (HCl) 1 M was purchased from LP Chemicals Ltd. (Cheshire, UK). Sodium chloride (NaCl) (MW 58.44 g/mol) was obtained from Fisher Scientific (Loughborough, UK).

### Pharma-ink preparation

For each printing test, 5 g of a mixture of drug and excipients was prepared. The individual components were mixed thoroughly in a mortar and pestle until homogenous, with the final optimised pharma-ink composition used for the 3DP of extended-release doxazosin printlets being: 95% w/w Klucel JF, 4% w/w D-mannitol, and 1% w/w doxazosin mesylate.

To determine the solid-state of the drug in the formulation without sensitivity issues, a 10% w/w doxazosin mesylate loaded pharma-ink was made, and the formulation was adjusted by reducing the Klucel JF quantity. The composition of this was: 86% w/w Klucel JF, 4% w/w D-mannitol, and 10% w/w doxazosin mesylate.

### Direct powder extrusion printing process

The chosen geometry for the first batch of printlets was cylindrical, with a varying width corresponding to the desired printlet mass and therefore the target doxazosin dose (Table 1). For the second batch of printlets, channels of varying diameter were introduced to ensure all printlets had the same surface area to volume (SA: V) ratio.

**Table 1:** Various cylindrical printlets printed in this study and their theoretical physical characteristics

Printlet	Height (mm)	Width (mm)	Channel diameter (mm)	SA: V ratio (mm^− 1^)	Theoretical mass (mg)	Theoretical dose (mg)
6 × 3.6	3.6	6	-	1.22	100	1
8 × 3.6	3.6	8	-	1.06	200	2
10 × 3.6	3.6	10	-	0.96	300	3
Ch 8 × 3.6	3.6	8	1.94	1.22	200	2
Ch 10 × 3.6	3.6	10	2.2	1.22	300	3

The prepared pharma-ink was added to the M3DIMAKER™ pharmaceutical 3D printer hopper (FABRX Ltd., London, UK), with a direct powder extrusion nozzle attachment. To design the printlets, AutoCAD 2014 (Autodesk Inc., CA, USA) was used to generate the stereolithography (.stl) file for the 3D printer software (Repetier Host Version 2.1.3., Nordrhein-Westfalen, Germany). The printer settings for the successful printlets in the Repetier Host software were: Rectilinear pattern, 100% infill, extrusion temperature of 170 °C, extrusion speed (20 mm/s), travel speed (90 mm/s), perimeter speed (60 mm/s), infill speed (20 mm/s), number of shells (2), and layer height (including the first layer) (0.4 mm).

### Thermal analysis

Thermogravimetric analysis (TGA) was used to characterise the individual components within the printed formulation, including the polymers, D-mannitol, and doxazosin. All samples (3–5 mg) were heated from 40 °C to 400 °C, at a heating rate of 10 °C/min in open aluminium pans using a Discovery TGA (TA instruments, Waters LLC, New Castle, DE, USA). The purge gas used was nitrogen, at a rate of 25 mL/min. All data was collected and analysed using the TA instruments Trios software (v. 4.5.0.5), and the % mass loss versus temperature was calculated.

Differential scanning calorimetry (DSC) was also used to characterise doxazosin, polymer, D-mannitol, pharma-ink and drug-loaded printlet. DSC measurements were performed using a Q2000 DSC (TA instruments, Waters LLC, New Castle, DE, USA). Calibration for cell constant and enthalpy was performed with indium (Tm = 156.6 °C, ΔHf = 28.71 J/g), based on the manufacturer’s instructions. The samples in TA aluminium pans and lids (Tzero) (3–5 mg) were heated to 300 °C, at a heating rate of 10 °C/min. Nitrogen gas at a flow rate of 50 mL/min was used to purge the system throughout. Data points were collected with the TA Advantage software for Q series (v. 2.8.394) and analysed using TA Instruments Universal Analysis 2000 (TA instruments-Waters LLC, New Castle, DE, USA).

### X-ray powder diffraction (XRPD) analysis

X-ray diffraction analysis was carried out with the Rigaku MiniFlex 600 (Rigaku, The Woodlands, TX, USA), equipped with a Cu Kα X-ray source of wavelength λ = 1.5418 Å. The x-ray angle range was 2θ = 3–40°, with a step size of 0.02° and a speed of 2°/min. The intensity was 15 mA, with an applied voltage of 40 kV. Analysis was carried out on the empty sample pan, pure drug and polymer powders, powder mixtures before printing, and printed samples which were 3D printed into a 23 mm diameter x 1 mm height disc to fit the sample pan.

### Fourier-transform infrared spectroscopy (FTIR) analysis

FTIR analysis of the printlets, pharma-inks, and individual formulation components (doxazosin, Klucel JF, and D-mannitol) was carried out on the Perkin Elmer Spectrum 100 FTIR with an attenuated total reflectance (ATR) accessory (Perkin Elmer, Buckinghamshire, UK). All samples were scanned in the wavenumber range of 650–4000 cm^− 1^, with 32 scans and a resolution of 4 cm^− 1^.

### Printlet morphology

Physical dimensions (height and diameter) of the printlets were measured using a digital calliper DIGIMAX^®^ (Kunststoffwerk AG Buchs, Buchs, Switzerland), and *n* = 10 printlets from each print were assessed.

### Scanning electron microscopy (SEM) imaging

The printlet segment were mounted onto a 3.2 × 8 mm aluminium disc mount and splutter-coated for 120 s with gold for visualisation purposes. Images of the polymer powders and printlet were taken with a JSM-840 A Scanning Microscope (JEOL GmbH, Freising, Germany).

### Printlet strength determination

The crushing strength of the smallest 6 × 3.6 mm printlets (*n* = 3) was measured using a traditional tablet hardness tester TBH 200 (Erweka GmbH, Heusenstamm, Germany), whereby an increasing force was applied perpendicular to the printlet axis to opposite sides of a printlet until the printlet fractured.

### Drug loading quantification

To create the doxazosin calibration curve, a specific mass of drug (100 mg) was dissolved in methanol until complete dissolution (*n* = 3). The stock solution was then titrated to make up the calibration samples, ranging from 0.004 to 0.1 mg/mL in concentration, which were then filtered through a 0.45 μm filter (Millipore Ltd., Carrigtwohill, Ireland). To quantify the doxazosin loading in each printlet, printlets from different printing batches were taken at random, weighed, and then dissolved in methanol until complete dissolution (*n* = 3). The solutions were then filtered through a 0.45 μm filter (Millipore Ltd., Carrigtwohill, Ireland).

The drug concentration was determined with High-Performance Liquid Chromatography-UV (HPLC-UV), using a Hewlett Packard 1260 Series HPLC system (Agilent Technologies, Stockport, UK). The HPLC analysis was performed at 25 °C, using a mobile phase of 60:40 v/v methanol: pH 4.8 phosphate buffer at a flow rate of 1.0 mL/min. 15 µL of sample was injected into a Zorbax Eclipse Plus C18 5 μm (150 × 4.6 mm) column (Agilent Technologies, Stockport, UK) and doxazosin was detected at a wavelength of 251 nm, with a retention time of approximately 5 min.

To make the pH 4.8 phosphate buffer for the HPLC mobile phase, 6.8 g of potassium dihydrogen orthophosphate was weighed and transferred to a 1000 mL volumetric flask. Deionised water was then added until 1000mL was reached. The solution was left to stir until fully dissolved, then the pH was adjusted with 5 M NaOH or 1 M HCl and finally filtered (Whatman qualitative grade 1 filter paper – Fisher Scientific Ltd., Loughborough, UK).

### In vitro dissolution testing

To assess the in vitro release profile of the doxazosin printlets a 708-DS USP II dissolution apparatus with a minipaddle set up was used (Agilent Technologies, Stockport, UK). The printlets (*n* = 3) were placed in small volume (300 mL) clear flat bottom glass dissolution tubes (27-5100) (Agilent Technologies, Stockport, UK) filled with 50 mL of simulated gastric fluid (SGF) (0.2% w/v NaCl dissolved in 1000 mL deionised water, followed by the addition of 1 M HCl to reduce the pH to 1.2), which was heated to 37 °C, and kept at a constant paddle speed of 75 rpm. 1 mL samples were removed at specific time intervals and replaced with 1 mL of fresh media which was also maintained at 37 °C. All samples were then filtered through 0.45 μm filters (Millipore Ltd., Carrigtwohill, Ireland), and analysed using HPLC-UV with the previously described method.

To compare the dissolution profiles of each printlet, a difference factor (f_1_) and similarity factor (f_2_) were calculated to determine the degree of similarity between each dissolution profile. Mathematical approaches previously proposed by Moore and Flanner [48], the following Eqs. (1 and 2) were used to calculate f_1_ and f_2_ for the two printlet batches:

1 $${f_1} = \left\{ {\frac{{\sum\nolimits_{t = 1}^n {\left| {Rt - Tt} \right|} }}{{\sum\nolimits_{t = 1}^n {Rt} }}} \right\} \times 100$$

2 $${f_2} = 50\,\log \left\{ {{{\left[ {1 + \left( {\frac{1}{n}} \right)\sum\nolimits_{t = 1}^n {{{\left( {Rt - Tt} \right)}^2}} } \right]}^{ - 0.5}} \times 100} \right\}$$

Where *n* is the number of time points, Rt is the average % drug release of the reference product at time t (printlet 6 × 3.6), and Tt is the average % drug release of the test product (all other printlets) at time t. Only one data point after 85% of drug release was included in the calculations.

An f_1_ value between 0 and 15 suggests that the dissolution profiles are similar, with a value of 0 corresponding to identical dissolution profiles and a value above 15 indicating no similarity between the profiles. The f_2_ value ranges from 0 to 100, with 100 corresponding to identical dissolution profiles. According to the Food and Drug Administration (FDA) and European Medicines Agency (EMA) guidelines, an f_2_ value between 50 and 100 suggests similarity between the dissolution profiles [49].

### Kinetic modelling

To determine the drug release kinetic model that best describes the extended-release profile of each printlet, various models were investigated by plotting the in vitro dissolution profiles to zero-order (Eq. 3), first order (Eq. 4), Korsmeyer-Peppas (Eq. 5), and Hixson-Crowell (Eq. 6) kinetic models. All data points were used except for the Korsmeyer-Peppas model, whereby the first ∼ 60% of drug release was plotted, as the model’s assumptions are most valid in the early stages of the release process. After adjustment using each equation, the R^2^ values were calculated to determine the doxazosin mesylate release mechanism from all printlets.

3 $${Q_t} = {Q_0} + {K_0}t$$

Where Q_t_ represents the quantity of drug released in time t (cumulative drug release), Q_0_ is the initial concentration of drug in the solution (generally, Q_0_ = 0), and K_0_ is the zero-order release constant, expressed in units of quantity/time.

4 $$\log \,{Q_t} = \log \,{Q_0} - \left( {\frac{{{K_1}t}}{{2.303}}} \right)$$

Where Q_t_ is the amount of drug released in time t (cumulative drug release), Q_0_ is the initial amount of drug dissolved, and K_1_ is the first-order rate constant.

5 $$\frac{{{M_t}}}{{{M_{\infty \:}}}} = {K_{KP}}{t^n}$$

Where M_t_/$\:{\text{M}}_{{\infty\:}}$ is the amount of drug released at time t, $\:{M}_{\infty\:}$ the total amount of drug in the dosage form, K_m_ is the release constant, and *n* is the release exponent.

6 $${Q_0}^{1/3} - {Q^{1/3}} = {K_{HC}}t$$

Where Q_0_ is the initial amount of drug in the solution (generally, Q_0_ = 0), Q is the amount of drug remaining at time t, and K_HC_ is the rate constant.

## Results and discussion

**Fig. 1:**
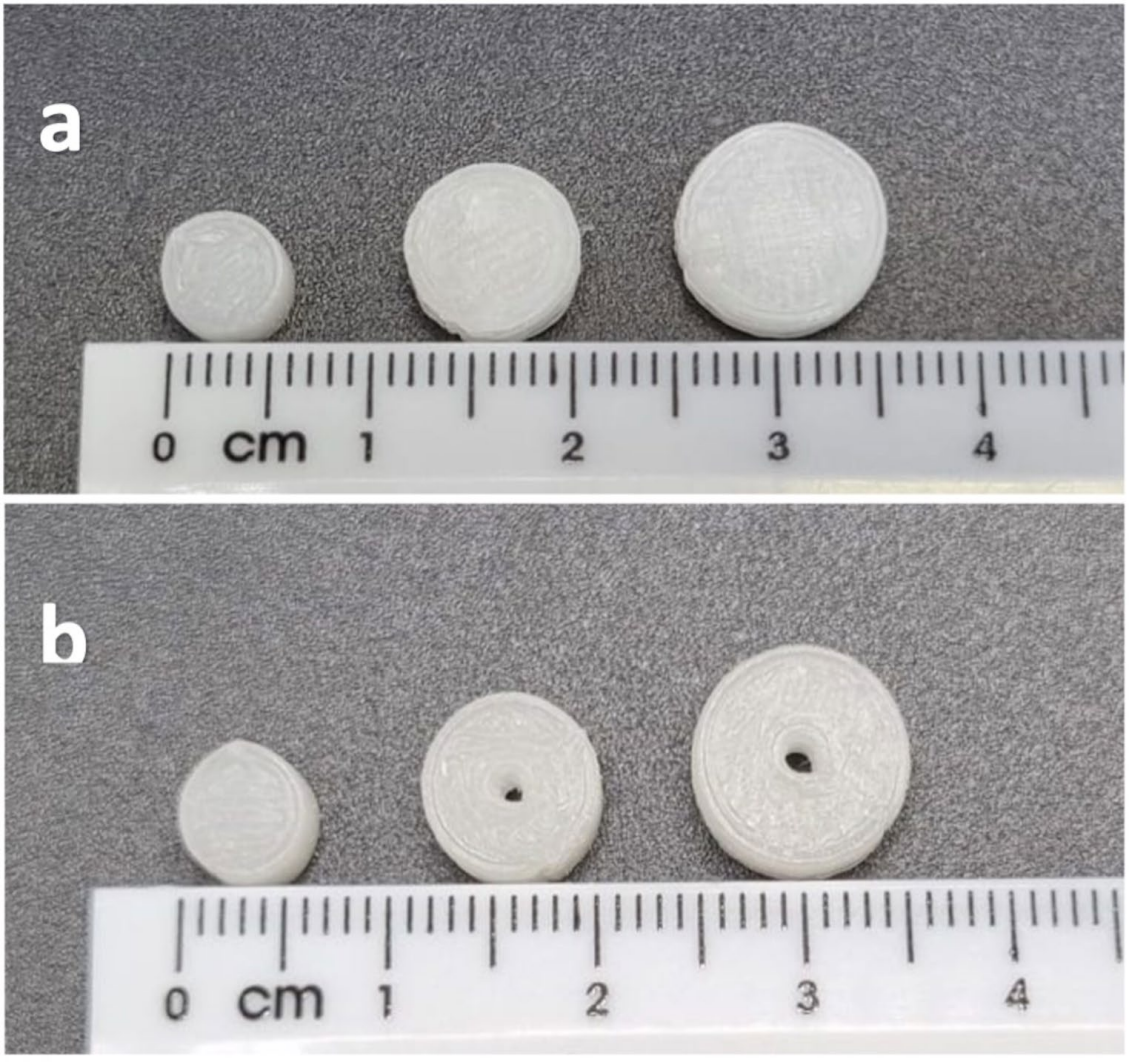
Various printlet sizes printed using the optimised pharma-ink: (**a**) From left to right: 6 × 3.6; 8 × 3.6; 10 × 3.6, (**b**) From left to right: 6 × 3.6; Ch 8 × 3.6; Ch 10 × 3.6

All printlets were of a high resolution and white in colour, with no discolouration during or after printing (Fig. 1). The final pharma-ink consisted of only three components: 94% w/w Klucel JF, 5% w/w D-mannitol, and 1% w/w doxazosin mesylate. This simple pharma-ink reduces the need for additional excipients such as fillers, binders, lubricants, or disintegrants, which are often needed in conventional tabletting processes [50]. With fewer formulation components, there is also a reduced risk of any potential interactions between excipients themselves or with the body, which are not always inert as investigated by numerous studies [51–55]. All printlets were printed from one batch of powder feedstock (pharma-ink), meaning dose titration would be much simpler and quicker for a pharmacist that must print the medication in the form of pharmaceutical compounding. The user would only have to adjust the software settings for the dimensions of the printlet, without changing the pharma-ink which would require completely changing the DPE printhead or cleaning the system.

**Fig. 2:**
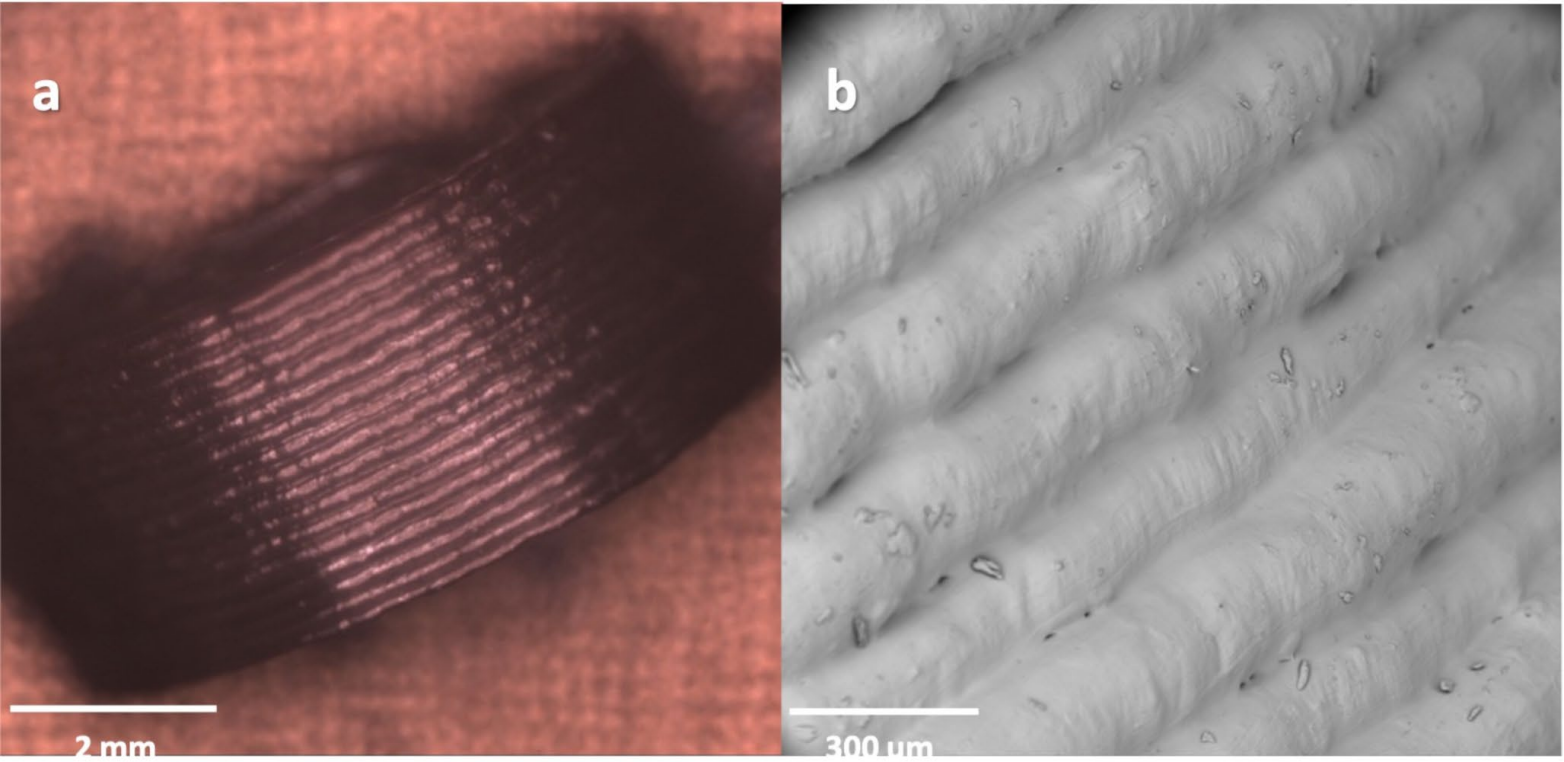
SEM images of (**a**) side view of all deposited layers in the printlet and (**b**) zoomed in view of each deposited layer

Based on the SEM images of the side of the printlet, the individual printed layers can be clearly seen, with no gaps or inconsistencies between the layers (Fig. 2). Consistent pharma-ink deposition results in a strong printlet, with a uniform density throughout. The high printlet mechanical strength was confirmed by the breaking force data, with the average breaking force of the smallest printlet (6 × 3.6 mm) being 483 ± 1 N, very close to the maximum value measurable by the hardness tester (500 N). To ensure a similar extended-release profile for all printlets, the absence of gaps during printing is also important, leading to a predictable drug release profile.

DPE 3DP successfully printed all cylindrical printlets with minimal variation in the height, width, and channel diameter where applicable (Table 2). All printlet dimensions were close to the theoretical dimensions previously shown in Table 1. The printlet masses showed more variation and larger SD values, which can be explained by the variability in the amount of material being pushed out of the printhead during printing. To alleviate the variation in printlet mass, a force feeder could be added to the DPE hopper, which would add an additional mixing feature to the system as the powder is heated to ensure a consistent flow through the screw. This has already been done by Rosch et al., whereby a wire was shaped into an open noose and added to the coupling element, constantly rotating to ensure the powder is moved during printing [56]. Since all printlets were printed from the same batch of pharma-ink powder feed, the drug loading was calculated by dissolving printlets at random from different batches. Based on three different printlets, the drug loading was found to be 0.97 ± 0.01% w/w, close to the theoretical loading of 1% w/w.

**Table 2:** Physical weight and dimensions of all channel and non-channel printlets (*n* = 10)

Printlet	Mass (mg)	Height (mm)	Width (mm)	Channel diameter (mm)
6 × 3.6	109.51 ± 7.35	3. 60 ± 0.10	6.04 ± 0.07	N/A
8 × 3.6	205.5 ± 10.30	3.62 ± 0.06	8.07 ± 0.05	N/A
10 × 3.6	303.15 ± 11.57	3.59 ± 0.06	10.08 ± 0.06	N/A
Ch 8 × 3.6	202.57 ± 8.32	3.69 ± 0.09	8.05 ± 0.08	1.95 ± 0.04
Ch 10 × 3.6	313.9 ± 8.78	3.65 ± 0.06	10.09 ± 0.16	2.16 ± 0.09

**Fig. 3:**
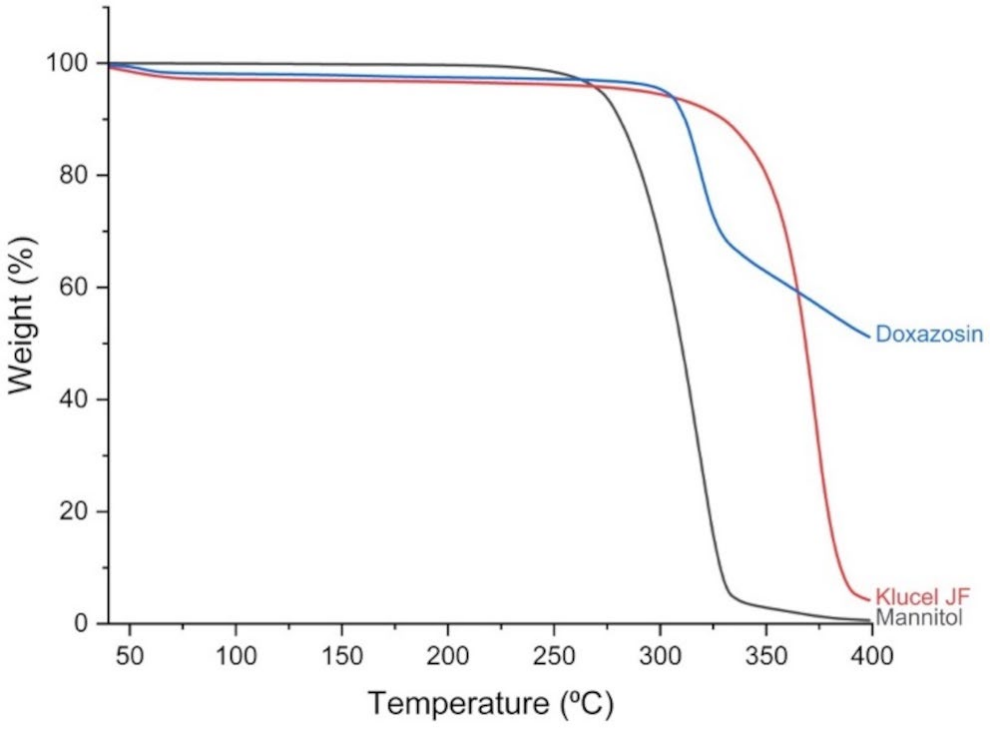
TGA thermogram of all pharma-ink components to assess degradation

To ensure no degradation has occurred during printing, TGA analysis was also conducted on all components of the pharma-ink (Fig. 3). D-mannitol exhibited the lowest degradation temperature onset of 239 °C, followed by doxazosin at 277 °C, and Klucel JF at 285 °C. Both Klucel JF and doxazosin have a ∼ 3% weight loss at around 80 °C, which is attributed to water evaporation, most likely due to some moisture presence in the samples. Printing of all printlets was carried out at 170 °C, which is well below the degradation temperatures of all components, confirming that DPE 3DP led to no drug or excipient degradation.

**Fig. 4:**
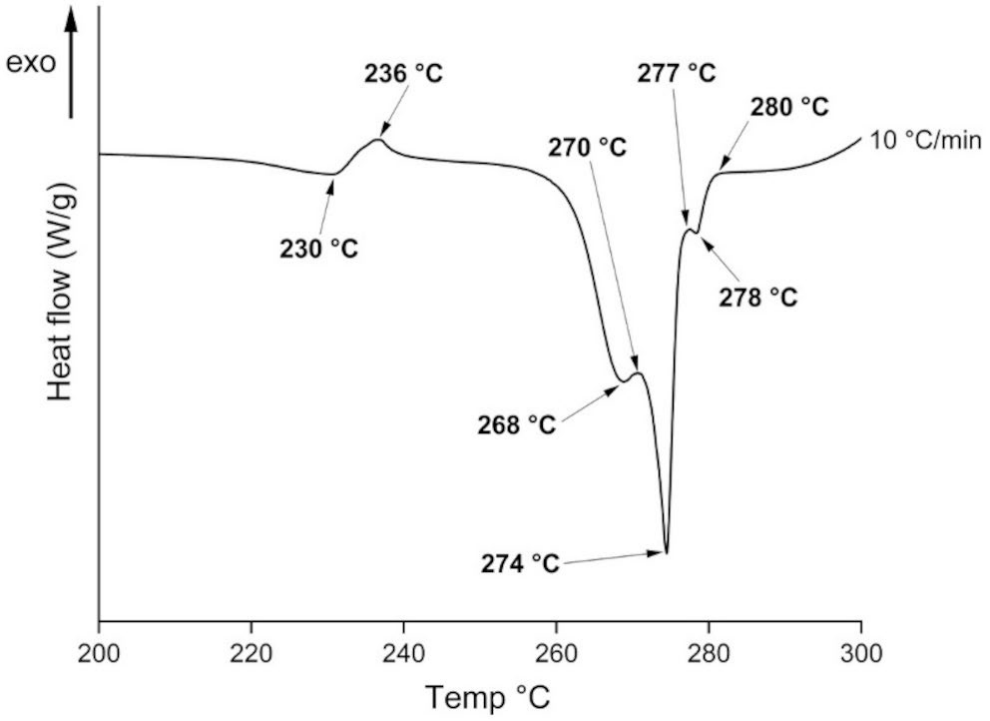
Zoomed in (200–300 °C) DSC thermogram of doxazosin mesylate and its mixture of polymorphs. All indicated temperatures are presented as T_max_

Differential scanning calorimetry (DSC) was first carried out to assess the solid-state structure of the API on its own, as it is known in the literature to contain many polymorphs (Fig. 4) [57–59]. When zoomed into the thermogram, many thermal events are taking place in the API sample. Consecutive melting (endothermic event) and crystallisation (exothermic event) occur at different temperatures, indicating that the sample has a mixture of five polymorphs. The first polymorph (and the least stable one) melts at a T_max_ of 230 °C, and recrystallises at a T_max_ of 236 °C. This is then followed by successive melting and recrystallisation of each polymorph within the sample, until the last polymorph melts and recrystallises into the most stable polymorph at T_max_ 280 °C. The first polymorph melting occurs at 230 °C, well above the printing temperature used (170 °C), meaning recrystallisation or melting should not occur during printing.

**Fig. 5:**
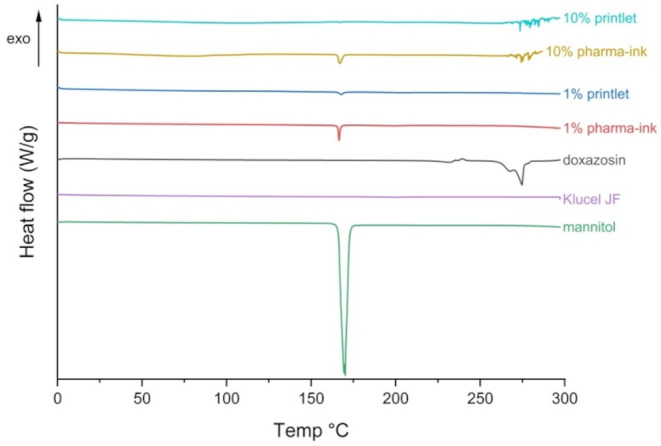
DSC thermogram of all pharma-ink components, as well as the 1% w/w and 10% w/w pharma-inks and printlets

Differential scanning calorimetry (DSC) was then carried out to assess the solid-state structure of the API in the pharma-ink before printing and in the printlets (Fig. 5). As the drug loading in the pharma-ink (1% w/w) is below the sensitivity of most analytical techniques, information on the solid-state structure of doxazosin mesylate was going to be difficult to assess. Therefore, another pharma-ink with a higher drug loading of 10% w/w was made, by reducing the Klucel JF component quantity but keeping the D-mannitol (4% w/w) the same. This would ensure that the drug loading is above the sensitivity of all analytical techniques, and it can be assumed that whatever changes occur to the higher drug loading printlet, also happen to the 1% w/w printlet.

When comparing the DSC thermograms of the 1% w/w pharma-ink and printlet, no distinct melting endotherms for the drug can be seen in both, as expected due to the sensitivity limit of the instrument. D-mannitol shows a sharp endothermic peak at T_max_ 167 °C, corresponding to its melting point which has also been confirmed by the literature [60]. This endotherm is present in all pharma-inks and printlets but is smaller, most likely due to the small quantity (4% w/w). The presence of D-mannitol endotherm in both printlets (1 and 10% w/w) also confirms that no degradation has occurred during printing. Despite increasing the doxazosin content to 10% w/w, no melting endotherm or crystallisation exotherm can be seen in the 10% pharma-ink or printlet. The first melting endotherm in the doxazosin sample at 230 °C is very small and may be masked by the large quantity of polymer in both the 10% w/w pharma-ink and printlet. In addition, the first polymorph melts at 230 °C but cannot recrystallise into a more stable form as it is now dispersed within the polymeric matrix, which is likely due to most of the formulation being made up of the polymer (86% w/w). Both the pharma-ink and printlet al.so exhibit noise at 275 °C, which is most likely degradation, confirmed by the TGA thermogram of doxazosin.

**Fig. 6:**
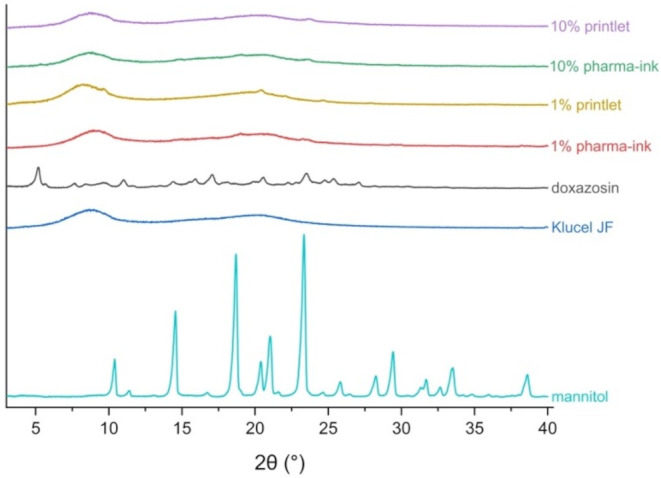
XRPD diffractograms of all pharma-ink components in the formulation, as well as the 1% and 10% w/w pharma-inks and printlets

Similarly to DSC, it was also difficult to assess the solid-state structure of doxazosin using the diffraction patterns of XRPD. As seen in Fig. 6, doxazosin does not have sharp intense peaks in the diffractogram. This means it cannot be distinguished from noise or other excipients in the formulation such as mannitol, which is made up of sharp and intense crystalline peaks. The diffraction peaks of doxazosin and mannitol also overlap at 14°, 21°, 23°, and at 25.5°, making it harder to isolate and detect any crystalline doxazosin specific peaks. This is evident in the 10% pharma-inks and printlets, where a small peak present in both the pharma-ink and printlet at 23° could correspond to doxazosin or mannitol. The most intense peak of doxazosin at 5° is also not seen in either the pharma-ink or printlet, giving no clear insight into the solid-state structure of doxazosin.

**Fig. 7:**
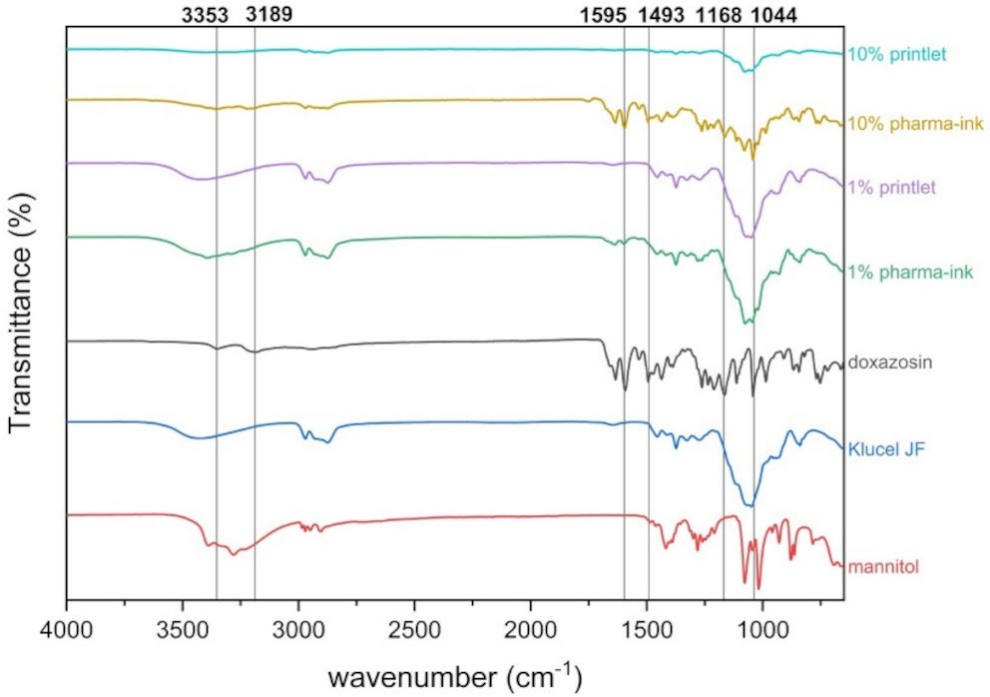
FTIR spectra of all pharma-ink components, as well as the 1% and 10% pharma-inks and printlets. Lines correspond to significant peaks in the crystalline doxazosin sample

On the other hand, FTIR analysis seemed to provide more information on the solid-state structure of doxazosin in the pharma-ink and printlet (Fig. 7). Crystalline doxazosin exhibits two broad peaks at 3357 and 3180 cm^− 1^, corresponding to the N-H stretching of the aromatic amine. Other characteristic drug peaks include the N-H amine bending at 1595 cm^− 1^, C-O stretching at 1168 cm^− 1^, and a sharp C-O peak at 1044 cm^− 1^ [61, 62]. Significant peaks do not overlap with the other components (Klucel JF and mannitol), making it easier to determine the solid-state structure of the drug. Again, 10% w/w pharma-ink and printlets were analysed and clear differences can be seen when compared to the 1% w/w formulations. Both the 1% w/w pharma-ink and printlet FTIR spectra look identical, again a result of the sensitivity of the technique. On the other hand, a clear difference can be seen between the 10% w/w pharma-ink and printlet. The two small N-H stretching peaks are present in the pharma-ink but disappear in the printlet, suggesting the drug is amorphous in the printlet. This is the case for all the characteristic peaks of doxazosin, which are present in the 10% w/w pharma-ink but are not visible in the 10% w/w printlet. This can be explained by the fact that amorphous materials often exhibit broader and less intense peaks, due to the lack of long-range order [63]. Therefore, some peaks present in the crystalline state may disappear in the amorphous form due to the disrupted molecular symmetry. Based on all the analytical date of the pharma-inks and printlets, it can be stated that the metastable polymorph of doxazosin is crystalline in the pharma-ink but becomes molecularly dispersed within the polymeric matrix during extrusion printing and forms an amorphous solid dispersion in the 1% w/w printlets.

**Fig. 8:**
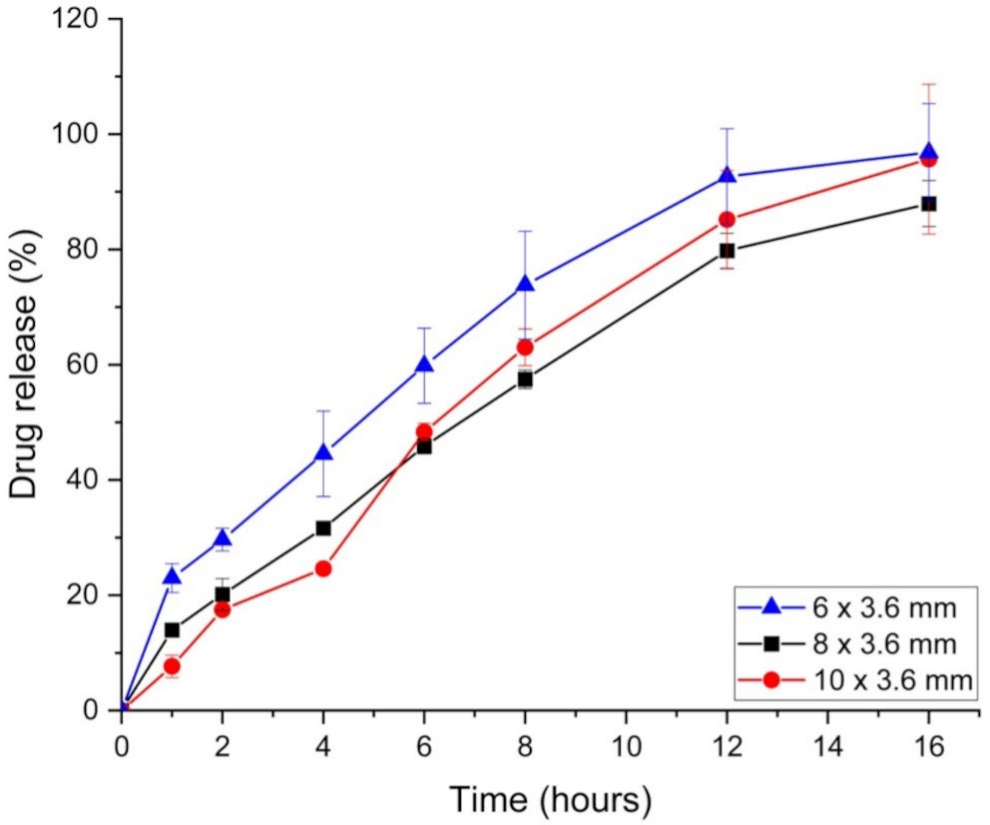
In vitro doxazosin release profiles of all printlets printed from the same batch with varying SA: V ratios (*n* = 3)

After printing, in vitro dissolution testing was conducted to assess the release profiles of each printlet. The three non-channel printlets, which have varying SA: V ratios (6 × 3.6–1.22 mm^− 1^; 8 × 3.6–1.06 mm^− 1^; 10 × 3.6–0.96 mm^− 1^) are expected to show varying release profiles. This is clear in the dissolution data (Fig. 8), where distinct differences are observed between the larger printlets (8 × 3.6 and 10 × 3.6) and the smallest printlet (6 × 3.6). Numerous studies have already evaluated the effect of SA: V ratio on the release profile of the drug in 3D printed dosage forms, showing that it is a critical parameter in the prediction of drug release [64–71]. However, all studies have been carried out on SSE or FDM 3DP, and currently no work has been done to assess its effect on DPE 3D printed formulations.

HPC is a swellable polymer, exhibiting typical hydrogel behaviour when in contact with a solution, with higher molecular weight HPCs showing greater swellability [72]. Printlets with a larger initial SA: V ratio (6 × 3.6) likely undergo more rapid and uniform swelling, exposing more surface area to the dissolution medium earlier on compared to larger printlets (10 × 3.6). In the first 60 min of dissolution, the smallest 6 × 3.6 printlet exhibits a burst release of doxazosin, as any drug present in the outer layer dissolves quickly. As the HPC swells and forms a gel layer over time, drug release slows and reaches a steady state, controlled by diffusion through the gel or erosion of the polymer. Tablets with smaller SA: V ratios (e.g. 10 × 3.6) may form thicker gel layers, which could slow the diffusion of doxazosin. In contrast, larger SA: V ratios (6 × 3.6) may result in thinner gel layers, leading to faster diffusion and dissolution. Despite differences in release kinetics, all formulations achieved ∼ 100% drug release within the experimental timeframe (16 h), suggesting that the SA: V ratio impacts the release kinetics but not the total drug dissolution.

To compare the release profiles more closely, f1 and f2 values were calculated for each printlet comparison. High f1 scores of 23.07 and 23.85 were obtained for the smallest printlet (6 × 3.6) when compared to the two larger non-channel printlets, 8 × 3.6 and 10 × 3.6, respectively (Table 3). An f1 score above 15 indicates that there are no similarities between the release profiles, which is consistent with the f2 scores. Both larger non-channel printlets had f2 values below 50 (44.77 and 43.52) when compared to the smallest printlet. An f2 value below 50 also corresponds to no similarities between the release profiles. In contrast, the f1 and f2 scores between the two larger non-channel printlets (8 × 3.6 vs. 10 × 3.6) were significantly better, with a low f1 score of 11.79 and a high f2 score of 63.86, meaning both release profiles are similar. This can be explained by the small difference of 0.1 mm^− 1^ in SA: V ratio, with the 8 × 3.6 being 1.06 mm^− 1^ and the 10 × 3.6 being 0.96 mm^− 1^. On the other hand, the SA: V ratio differences are larger for the larger printlets vs. 6 × 3.6 (Table 1), explaining the high f1 score and low f2 score.

**Table 3:** The calculated difference (f1) and similarity (f2) scores of all printlet release profiles against each other

Printlets	f1 score	f2 score
6 × 3.6 vs. 8 × 3.6	23.07	44.77
6 × 3.6 vs. 10 × 3.6	23.85	43.52
8 × 3.6 vs. 10 × 3.6	11.79	63.86
6 × 3.6 vs. Ch 8 × 3.6	16.53	50.61
6 × 3.6 vs. Ch 10 × 3.6	18.58	48.26
Ch 8 × 3.6 vs. Ch 10 × 3.6	6.54	73.95

**Fig. 9:**
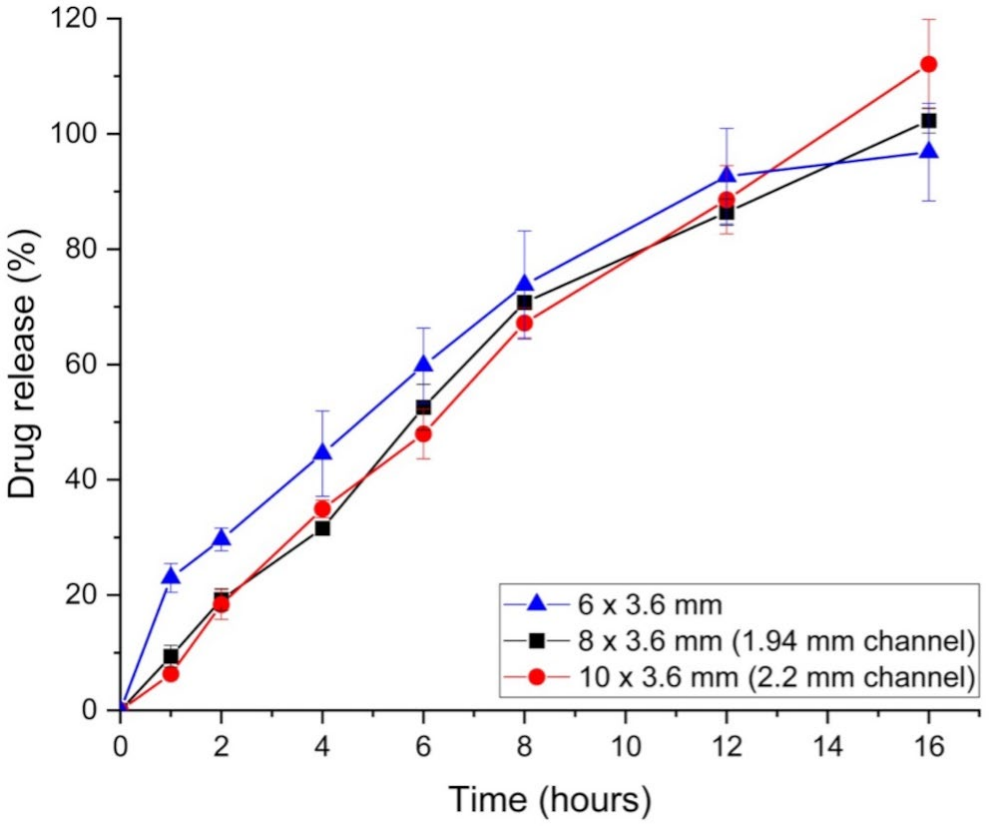
Drug release profiles of all printlets printed from the same batch with the same SA: V ratio through the introduction of channels (*n* = 3)

To improve the similarity between the drug release profiles of all printlets from a single printing batch, single channels of varying sizes were introduced in the centre of the larger printlets. These channels were designed to achieve the same theoretical SA: V ratio (1.22 mm^− 1^) as the smallest printlet (Table 1). This modification led to improved alignment of the release profiles across all three printlets (Fig. 9), as evidenced by improved similarity indices in the f1 and f2 scores for all pairwise printlet comparisons (Table 3). The most significant improvement was observed between the larger 8 × 3.6 and 10 × 3.6 printlets, where the f2 score increased from 63.86 to 73.95, and the f1 score decreased from 11.79 to 6.54.

Although all printlets has the same theoretical SA: V ratio, the channel printlets exhibited slightly slower dissolution compared to the smallest 6 × 3.6 printlet. Notably, the channel printlets did not exhibit the burst release of doxazosin observed in the first 60 min for the smallest printlet. However, during the later stages of dissolution (240 min and beyond), all three printlets showed similar steady-state release profiles, outperforming the non-channel printlets manufactured before.

The absence of a burst release in the channel printlets can be attributed to the channels facilitating a more controlled hydration process. This likely allowed for a quicker and more uniform formation of the gel layer surrounding the printlet, thereby moderating drug release. Initially, the dissolution media may preferentially penetrate through the channel, slowing overall drug release across the matrix. In contrast, the smallest non-channel printlet (6 × 3.6) exhibited less controlled hydration, leading to delayed gel layer formation and therefore an initial burst release of the drug. Once the gel layer was formed for all three printlets, the drug release rate stabilised, and their in vitro release profiles converged, ultimately resulting in comparable overall release patterns.

While the f2 scores for both channel printlets improved (Table 3), they remained close to 50, which is the threshold for indicating similarity between release profiles. An f2 score near 50 indicates an average difference of ∼ 10% between the release profiles. This variation could be influenced by differences in density among the printlets or limitations in the overall printing resolution. Extrusion-based 3DP techniques typically exhibit lower resolution when compared to other technologies, such as resin-based printing or selective laser sintering. Resolution in extrusion-based systems is largely determined by the nozzle diameter [73, 74]. To further improve the f1 and f2 scores for the channel printlets (Fig. 9), a smaller nozzle diameter of 0.2 mm could be used to ensure a higher printing resolution and more accurate reproduction of the intended SA: V ratios.

Various drug release kinetic models were applied to the in vitro drug release profiles to determine the best-fitting release mechanism for the doxazosin printlets (Table S1). Among the non-channel printlets, the Hixson-Crowell model provided the best fit. This model assumes that dissolution occurs in planes parallel to the surface of the dosage form, with the surface area decreasing proportionally over time while the geometry remains constant [75, 76]. This correlates well with the uniform cylindrical structure of the non-channel printlets, which promotes consistent surface dissolution.

The Korsmeyer-Peppas model also demonstrated a strong fit for the non-channel printlets, with high R^2^ values close to 1. This model describes drug release from polymeric matrices, such as a hydrogel, where multiple mechanisms occur, such as diffusion, swelling, and erosion [77]. Notably, the differing SA: V ratios among the three non-channel printlets result in distinct release exponents (n) within the Korsmeyer-Peppas model (Table S2). For the cylindrical geometry of the smaller 6 × 3.6 and 8 × 3.6 printlets, the release mechanism corresponds to anomalous transport (0.45 < *n* < 0.89), indicating a combination of drug diffusion and polymer swelling [78]. In contrast, the largest printlet exhibits super case II transport (*n* > 0.89), where drug release is primarily driven by extensive polymer relaxation or swelling. The difference between these correspond to the differences in size and SA: V ratio, as a larger printlet has a greater volume and therefore a thicker polymer matrix, meaning there is a slower diffusion driven release.

The channel-containing printlets also showed a strong correlation with the Korsmeyer-Peppas model, achieving high R^2^ values of 0.9937 and 0.9834 (Table S1). Both Ch 8 × 3.6 and Ch 10 × 3.6 exhibited super case II transport (Table S2), attributed to the faster gel layer formation discussed previously, as well as enhanced hydration and greater polymer relaxation facilitated by the presence of channels. Differently to the non-channel printlets, both channel printlets do not fit well with the Hixson-Crowell model. The channels disrupt the overall symmetry of the printlets, leading to dynamic and irregular changes in total surface area during dissolution.

3DP of medicines can address sex-based differences in dosing and drug therapies by enabling highly customisable and personalised medications. As shown in this study, tailored dosages can be manufactured for each sex, accounting for the fact that males and females metabolise drugs differently due to variations in body weight, fat composition, enzyme activity, and hormone levels. With only modifications in the printing software, 3DP allows precise control over the amount of API in each dosage form. Sex differences are also shown to occur in the presence of some commonly used excipients in pharmaceutical formulations. 3DP can modify the formulation to minimise the use of non-inert excipients, reducing the risk of side effects or differences in drug absorption between individuals. In the future, personalised medicine from 3DP can integrate sex-based data from pharmacological research to refine formulations further. This approach ensures that medicine development addresses both male and female physiological needs effectively.

## Conclusions

This work explored the capabilities of a novel extrusion-based 3DP technology, DPE 3DP, for fabricating extended-release doxazosin mesylate printlets with varying doses from a single batch of pharma-ink (powder feedstock). Successful printlets in varying doses (1 mg, 2 mg, and 3 mg) with low variability in mass and dimensions were printed from one batch of pharma-ink, demonstrating the potential of the technology to achieve dose titration by adjusting the printlet mass through its dimensions. Several analytical techniques were used to determine the solid-state of the drug in the formulation, with Fourier transform infrared spectroscopy confirming the crystalline nature of doxazosin in the pharma-ink, which then becomes molecularly dispersed within the polymeric matrix during heating and extrusion. In vitro dissolution testing revealed extended-release profiles, releasing ∼ 100% of the drug within 16 h. Differences in the SA: V ratios among the cylindrical printlets led to variations in drug release profiles, as confirmed by low f2 similarity scores (> 50). To standardise the theoretical SA: V ratios, central channels were introduced into the larger printlets. This modification resulted in more comparable in vitro dissolution profiles across the different printlets, demonstrating the feasibility of tailoring geometry to achieve uniform drug release behaviour.

## Electronic supplementary material

Below is the link to the electronic supplementary material.

Supplementary Material 1

## Data Availability

The datasets generated during and/or analysed during the current study are available from the corresponding author on reasonable request.

## References

[1] Clayton JA. Studying both sexes: a guiding principle for biomedicine. FASEB J. 2016;30(2):519–24. 26514164 10.1096/fj.15-279554 PMC4714546

[2] Rademaker M. Do women have more adverse drug reactions?? Am J Clin Dermatol. 2001;2(6):349–51. 11770389 10.2165/00128071-200102060-00001

[3] Madla CM et al. Let’s talk about sex: differences in drug therapy in males and females. Adv Drug Deliv Rev, 2021: p. 113804. 10.1016/j.addr.2021.05.014 34015416

[4] Faubion SS, et al. Statin therapy: does sex matter? Menopause. 2019;26(12):1425–35. 31513091 10.1097/GME.0000000000001412 PMC7664983

[5] Ochs HR, et al. Diazepam kinetics in relation to age and sex. Pharmacology. 1981;23(1):24–30. 7312934 10.1159/000137524

[6] Roehrs TA, Roth T. Gender differences in the efficacy and safety of chronic nightly Zolpidem. J Clin Sleep Med. 2016;12(3):319–25. 26446253 10.5664/jcsm.5574 PMC4773634

[7] Colombo D, et al. The GENDER ATTENTION observational study: gender and hormonal status differences in the incidence of adverse events during cyclosporine treatment in psoriatic patients. Adv Ther. 2017;34(6):1349–63. 28432647 10.1007/s12325-017-0526-7 PMC5487861

[8] Rathore SS, Wang Y, Krumholz HM. Sex-based differences in the effect of Digoxin for the treatment of heart failure. N Engl J Med. 2002;347(18):1403–11. 12409542 10.1056/NEJMoa021266

[9] Zucker I, Prendergast BJ. Sex differences in pharmacokinetics predict adverse drug reactions in women. Biol Sex Differ. 2020;11(1):32. 32503637 10.1186/s13293-020-00308-5 PMC7275616

[10] Oparil S, et al. Hypertens Nat Reviews Disease Primers. 2018;4(1):18014. 10.1038/nrdp.2018.14 PMC6477925 29565029

[11] Tapela N, et al. Prevalence and determinants of hypertension control among almost 100 000 treated adults in the UK. Open Heart. 2021;8(1):e001461. 33707223 10.1136/openhrt-2020-001461 PMC7957140

[12] Carey RM, et al. Prevention and control of hypertension: JACC health promotion series. J Am Coll Cardiol. 2018;72(11):1278–93. 30190007 10.1016/j.jacc.2018.07.008 PMC6481176

[13] Wykretowicz A, et al. Add-on therapy with Doxazosin in patients with hypertension influences arterial stiffness and albuterol-mediated arterial vasodilation. Br J Clin Pharmacol. 2007;64(6):792–5. 17635498 10.1111/j.1365-2125.2007.02980.x PMC2198780

[14] Wykretowicz A, Guzik P, Wysocki H. Doxazosin in the current treatment of hypertension. Expert Opin Pharmacother. 2008;9(4):625–33. 18312163 10.1517/14656566.9.4.625

[15] Calvo C, et al. Doxazosin GITS versus standard Doxazosin in mild to moderate hypertension. Int J Cardiol. 2005;101(1):97–104. 15860390 10.1016/j.ijcard.2004.07.005

[16] Chung M, et al. Clinical pharmacokinetics of Doxazosin in a controlled-release Gastrointestinal therapeutic system (GITS) formulation. Br J Clin Pharmacol. 1999;48(5):678–87. 10594469 10.1046/j.1365-2125.1999.00067.x PMC2014349

[17] *Food and Drug Administration Centre for Drug Evaluation and Research. Clinical Pharmacology and Biopharmaceutics Review(s)*. 2005:21–269; Available from: https://www.accessdata.fda.gov/drugsatfda_docs/nda/2005/021269s000_ClinPharmR.pdf

[18] Vaz VM, Kumar L. 3D printing as a promising tool in personalized medicine. AAPS PharmSciTech. 2021;22(1):49. 33458797 10.1208/s12249-020-01905-8 PMC7811988

[19] Englezos K, et al. 3D printing for personalised medicines: implications for policy and practice. Int J Pharm. 2023;635:122785. 36849040 10.1016/j.ijpharm.2023.122785

[20] Seoane-Viaño I, et al. Translating 3D printed pharmaceuticals: from hype to real-world clinical applications. Adv Drug Deliv Rev. 2021;174:553–75. 33965461 10.1016/j.addr.2021.05.003

[21] Narala S, et al. 3D printing in vaginal drug delivery: a revolution in pharmaceutical manufacturing. Expert Opin Drug Deliv. 2024;21(11):1543–57. 38236621 10.1080/17425247.2024.2306139

[22] Denis L et al. Developing an innovative 3D printing platform for production of personalised medicines in a hospital for the OPERA clinical trial. Int J Pharm, 2024: p. 124306. 10.1016/j.ijpharm.2024.124306 38871137

[23] Placone JK, Engler AJ. Recent advances in Extrusion-Based 3D printing for biomedical applications. Adv Healthc Mater. 2018;7(8):e1701161. 29283220 10.1002/adhm.201701161 PMC5954828

[24] Zhang B, et al. Development of combi-pills using the coupling of semi-solid syringe extrusion 3D printing with fused deposition modelling. Int J Pharm. 2022;625:122140. 36031167 10.1016/j.ijpharm.2022.122140

[25] Algahtani MS, Mohammed AA, Ahmad J. Extrusion-Based 3D printing for pharmaceuticals: contemporary research and applications. Curr Pharm Des. 2018;24(42):4991–5008. 30636584 10.2174/1381612825666190110155931

[26] Azad MA et al. Polymers for Extrusion-Based 3D printing of pharmaceuticals: A holistic Materials-Process perspective. Pharmaceutics, 2020. 12(2). 10.3390/pharmaceutics12020124 PMC7076526 32028732

[27] Auriemma G, et al. Additive manufacturing strategies for personalized drug delivery systems and medical devices: fused filament fabrication and semi solid extrusion. Molecules. 2022;27. 10.3390/molecules27092784 10.3390/molecules27092784 PMC9100145 35566146

[28] Rodríguez-Pombo L, et al. 3D printed personalized therapies for pediatric patients affected by adrenal insufficiency. Expert Opin. Drug Deliv.; 2024. 10.1080/17425247.2024.2399706 39268761

[29] Rodríguez-Pombo L, et al. Paediatric clinical study of 3D printed personalised medicines for rare metabolic disorders. Int J Pharm. 2024;657:124140. 38643809 10.1016/j.ijpharm.2024.124140

[30] Rodríguez-Maciñeiras X, et al. Advancing medication compounding: use of a pharmaceutical 3D printer to auto-fill Minoxidil capsules for dispensing to patients in a community pharmacy. Int J Pharm. 2025;671:125251. 39863027 10.1016/j.ijpharm.2025.125251

[31] Cui M, et al. Opportunities and challenges of three-dimensional printing technology in pharmaceutical formulation development. Acta Pharm Sinica B. 2021;11(8):2488–504. 10.1016/j.apsb.2021.03.015 PMC8447232 34567958

[32] Seoane-Viaño I, et al. Semi-solid extrusion 3D printing in drug delivery and biomedicine: personalised solutions for healthcare challenges. J Controlled Release. 2021;332:367–89. 10.1016/j.jconrel.2021.02.027 33652114

[33] Iqbal H et al. Status of polymer fused deposition modeling (FDM)-Based Three-Dimensional printing (3DP) in the pharmaceutical industry. Polym (Basel), 2024. 16(3). 10.3390/polym16030386 PMC10857269 38337275

[34] Elbadawi M et al. M3DISEEN: A novel machine learning approach for predicting the 3D printability of medicines. Int J Pharm, 2020: p. 119837. 10.1016/j.ijpharm.2020.119837 32961295

[35] Hoffmann L, Breitkreutz J, Quodbach J. Fused deposition modeling (FDM) 3D printing of the Thermo-Sensitive peptidomimetic drug Enalapril maleate. Pharmaceutics, 2022. 14(11). 10.3390/pharmaceutics14112411 PMC9695326 36365230

[36] Abdella S, et al. 3D printing of Thermo-Sensitive drugs. Pharmaceutics. 2021;13. 10.3390/pharmaceutics13091524 10.3390/pharmaceutics13091524 PMC8468559 34575600

[37] Goyanes A, et al. Direct powder extrusion 3D printing: fabrication of drug products using a novel single-step process. Int J Pharm. 2019;567:118471. 31252147 10.1016/j.ijpharm.2019.118471

[38] Zheng Y, et al. Melt extrusion deposition (MED™) 3D printing technology – A paradigm shift in design and development of modified release drug products. Int J Pharm. 2021;602:120639. 33901601 10.1016/j.ijpharm.2021.120639

[39] Mora-Castaño G, et al. Optimising 3D printed medications for rare diseases: In-line mass uniformity testing in direct powder extrusion 3D printing. Int J Pharm. 2025;668:124964. 39557179 10.1016/j.ijpharm.2024.124964

[40] Fanous M, et al. Simplification of fused deposition modeling 3D-printing paradigm: feasibility of 1-step direct powder printing for immediate release dosage form production. Int J Pharm. 2020;578:119124. 32035253 10.1016/j.ijpharm.2020.119124

[41] Mendibil X et al. Direct powder extrusion of Paracetamol loaded mixtures for 3D printed pharmaceutics for personalized medicine via low temperature thermal processing. Pharmaceutics, 2021. 13(6). 10.3390/pharmaceutics13060907 PMC8234073 34205280

[42] Boniatti J et al. Direct powder extrusion 3D printing of praziquantel to overcome neglected disease formulation challenges in paediatric populations. Pharmaceutics, 2021. 13(8). 10.3390/pharmaceutics13081114 PMC8398999 34452075

[43] Ong JJ, et al. 3D printed opioid medicines with alcohol-resistant and abuse-deterrent properties. Int J Pharm. 2020;579:119169. 32087263 10.1016/j.ijpharm.2020.119169

[44] Sánchez-Guirales SA et al. Understanding direct powder extrusion for fabrication of 3D printed personalised medicines: A case study for Nifedipine minitablets. Pharmaceutics, 2021. 13(10). 10.3390/pharmaceutics13101583 PMC8537449 34683875

[45] Pistone M, et al. Direct cyclodextrin-based powder extrusion 3D printing for one-step production of the BCS class II model drug niclosamide. Drug Delivery Translational Res. 2022;12(8):1895–910. 10.1007/s13346-022-01124-7 PMC9242976 35138629

[46] Pistone M, et al. Direct cyclodextrin based powder extrusion 3D printing of Budesonide loaded mini-tablets for the treatment of eosinophilic colitis in paediatric patients. Int J Pharm. 2023;632:122592. 36626971 10.1016/j.ijpharm.2023.122592

[47] Krueger L, et al. Clinical translation of 3D printed pharmaceuticals. Nat Reviews Bioeng. 2024;2(10):801–3.

[48] Moore JW, Flanner HH. Mathematical comparison of dissolution profiles. Pharm Technol. 1996;20(6):64–74.

[49] Muselík J et al. A critical overview of FDA and EMA statistical methods to compare in vitro drug dissolution profiles of pharmaceutical products. Pharmaceutics, 2021. 13(10). 10.3390/pharmaceutics13101703 PMC8539859 34683995

[50] Kestur U, Desai D. Excipients for Conventional Oral Solid Dosage Forms, in Pharmaceutical Excipients. 2016:51–95.

[51] Amaral Silva D, Löbenberg R, Davies NM. Are excipients inert?? Phenytoin pharmaceutical investigations with new incompatibility insights. J Pharm Pharm Sci. 2018;21(1s):29745. 29702046 10.18433/jpps29745

[52] Panakanti R, Narang AS. Impact of excipient interactions on drug bioavailability from solid dosage forms. Pharm Res. 2012;29(10):2639–59. 22610283 10.1007/s11095-012-0767-8

[53] Fabiano V, Mameli C, Zuccotti GV. Paediatric pharmacology: remember the excipients. Pharmacol Res. 2011;63(5):362–5. 21241804 10.1016/j.phrs.2011.01.006

[54] Bobillot M, et al. Potentially harmful excipients: state of the Art for oral liquid forms used in neonatology and pediatrics units. Pharmaceutics. 2024;16. 10.3390/pharmaceutics16010119 10.3390/pharmaceutics16010119 PMC10820197 38258129

[55] Mai Y, et al. Sex-specific effects of excipients on oral drug bioavailability. Int J Pharm. 2022;629:122365. 36336203 10.1016/j.ijpharm.2022.122365

[56] Rosch M, et al. Development of an immediate release excipient composition for 3D printing via direct powder extrusion in a hospital. Int J Pharm. 2023;643:123218. 37467818 10.1016/j.ijpharm.2023.123218

[57] Gonzaga EV, et al. Doxazosin Free-Base structure determination and its equilibrium solubility compared to polymorphic Doxazosin mesylate forms A and H. Cryst Growth Des. 2019;19(2):737–46.

[58] Sohn Y-T, Lee Y-H. Polymorphism of Doxazosin mesylate. Arch Pharm Res. 2005;28(6):730–5. 16042084 10.1007/BF02969365

[59] Grčman M, Vrečer F, Meden A. Some Physico-chemical properties of Doxazosin mesylate polymorphic forms and its amorphous state. J Therm Anal Calorim. 2002;68(2):373–87.

[60] Mojiri A, et al. D-mannitol for medium temperature thermal energy storage. Sol Energy Mater Sol Cells. 2018;176:150–6.

[61] Gonçalves I, et al. A COMPLETE NMR STUDY OF DOXAZOSIN CHARACTERIZATION. Drug Anal Res. 2017;1:56–60.

[62] Al Ashmawy AZ, et al. New approach for administration of Doxazosin mesylate: comparative study between liquid and solid Self-nanoemulsifying drug delivery systems production and hosted by. Int J Res Pharm Sci. 2021;12:1095–101.

[63] Kaushal AM, Chakraborti AK, Bansal AK. FTIR studies on differential intermolecular association in crystalline and amorphous States of structurally related Non-Steroidal Anti-Inflammatory drugs. Mol Pharm. 2008;5(6):937–45. 19434918 10.1021/mp800098d

[64] Annuryanti F, et al. Fabrication and characterisation of 3D-Printed triamcinolone Acetonide-Loaded Polycaprolactone-Based ocular implants. Pharmaceutics. 2023;15. 10.3390/pharmaceutics15010243 10.3390/pharmaceutics15010243 PMC9863928 36678872

[65] Goyanes A, et al. Effect of geometry on drug release from 3D printed tablets. Int J Pharm. 2015;494(2):657–63. 25934428 10.1016/j.ijpharm.2015.04.069

[66] McDonagh T, Belton P, Qi S. An investigation into the effects of geometric scaling and pore structure on drug dose and release of 3D printed solid dosage forms. Eur J Pharm Biopharm. 2022;177:113–25. 35779743 10.1016/j.ejpb.2022.06.013

[67] Windolf H, Chamberlain R, Quodbach J. Dose-independent drug release from 3D printed oral medicines for patient-specific dosing to improve therapy safety. Int J Pharm. 2022;616:121555. 35131358 10.1016/j.ijpharm.2022.121555

[68] Kim YJ et al. Geometry-Driven fabrication of Mini-Tablets via 3D printing: correlating release kinetics with polyhedral shapes. Pharmaceutics, 2024. 16(6). 10.3390/pharmaceutics16060783 PMC11207496 38931904

[69] Patel P, Jinugu ME, Thareja P. Rheology and extrusion printing of κ-Carrageenan/Olive oil emulsion gel tablets with varying surface area to volume ratios for release of vitamin C and Curcumin. Langmuir. 2024;40(31):16069–84. 39058356 10.1021/acs.langmuir.4c00894

[70] Khaled SA, et al. Extrusion 3D printing of Paracetamol tablets from a single formulation with tunable release profiles through control of tablet geometry. AAPS PharmSciTech. 2018;19(8):3403–13. 30097806 10.1208/s12249-018-1107-z PMC6848047

[71] Reddy DP, R. and, Sharma V. Investigations of hybrid infill pattern in additive manufactured tablets: A novel approach towards tunable drug release. J Biomed Mater Res B Appl Biomater. 2023;111(11):1869–82. 37294096 10.1002/jbm.b.35290

[72] Borandeh S, et al. Polymeric drug delivery systems by additive manufacturing. Adv Drug Deliv Rev. 2021;173:349–73. 33831477 10.1016/j.addr.2021.03.022

[73] Januskaite P, et al. I spy with my little eye: A paediatric visual preferences survey of 3D printed tablets. Pharmaceutics. 2020;12(11):1100. 33212847 10.3390/pharmaceutics12111100 PMC7698452

[74] Aguilar-de-Leyva Á et al. 3D printing direct powder extrusion in the production of drug delivery systems: state of the Art and future perspectives. Pharmaceutics. 2024;16(4). 10.3390/pharmaceutics16040437 PMC11054165 38675099

[75] Paarakh P et al. Release kinetics-concepts and applications. 2018.

[76] Hixson AW, Crowell JH. Dependence of reaction velocity upon surface and agitation. Industrial Eng Chem. 1931;23(8):923–31.

[77] Korsmeyer RW, et al. MECHANISMS OF SOLUTE RELEASE FROM POROUS HYDROPHILIC POLYMERS. Int J Pharm. 1983;15(1):25–35.

[78] *5 - Mathematical models of drug release, in Strategies to Modify the Drug Release from Pharmaceutical Systems*, M.L. Bruschi, Editor. 2015, Woodhead Publishing. p. 63-86.

