# Supplementary material for: Sex-specific formulations of doxazosin mesylate via direct powder extrusion 3D printing
Source: Drug Deliv Transl Res. 2025 Apr 22;15(9):3338–50. doi: 10.1007/s13346-025-01862-4 (PMC12350568; doi:10.1007/s13346-025-01862-4)
Supplement: Supplementary file 1 — Supplementary Material 1 [file 13346_2025_1862_MOESM1_ESM.docx]

**Sex-specific formulations of doxazosin mesylate via direct powder extrusion 3D printing**

Patricija Januskaite^1^, Alvaro Goyanes^1,2,3,4*^, Mine Orlu^1*^, Abdul W. Basit^1,2*^

^1^Department of Pharmaceutics, UCL School of Pharmacy, University College London, 29-39 Brunswick Square, London, WC1N 1AX, UK.

^2^FABRX Ltd., Henwood House, Henwood, Ashford, Kent, TN24 8DH, UK.

^3^FABRX Artificial Intelligence, Calle Enrique Vidal Abascal 7, 15702, Santiago de Compostela, Spain.

^4^Departamento de Farmacología, Farmacia y Tecnología Farmacéutica, I+D Farma (GI-1645), Facultad de Farmacia, Instituto de Materiales (iMATUS) and Health Research Institute of Santiago de Compostela (IDIS), Universidade de Santiago de Compostela, 15782 Santiago de Compostela, Spain.

***Corresponding authors:** Alvaro Goyanes, PhD. FABRX Ltd., Henwood House, Henwood, Ashford, Kent, TN24 8DH, UK. Email address: a.goyanes@fabrx.co.uk. Mine Orlu, PhD. Department of Pharmaceutics, UCL School of Pharmacy, University College London, 29-39 Brunswick Square, London, WC1N 1AX, UK. Email address: [m.orlu@ucl.ac.uk](mailto:m.orlu@ucl.ac.uk). Abdul Basit, PhD. Department of Pharmaceutics, UCL School of Pharmacy, University College London, 29-39 Brunswick Square, London, WC1N 1AX, UK. Email address: [a.basit@ucl.ac.uk](mailto:a.basit@ucl.ac.uk)

| Table S1. R^2^ results for the various fitted drug release models of the doxazosin printlets. | | | | |  |
| --- | --- | --- | --- | --- | --- |
| **Printlet** | **Zero-order** | **First-order** | **Korsmeyer-Peppas** | **Hixson-Crowell** | |
| 6 x 3.6 | 0.9175 | 0.9772 | 0.9814 | 0.9906 | |
| 8 x 3.6 | 0.9685 | 0.9846 | 0.9894 | 0.9940 | |
| 10 x 3.6 | 0.9696 | 0.9524 | 0.9758 | 0.9885 | |
| Ch 8 x 3.6 | 0.9669 | 0.7774 | 0.9937 | 0.8587 | |
| Ch 10 x 3.6 | 0.9896 | 0.7863 | 0.9834 | 0.8224 | |

| Table S2. Release exponent results for all doxazosin printlets from the Korsmeyer-Peppas model, describing the release mechanism of the dosage forms in vitro. | | |
| --- | --- | --- |
| **Printlet** | **Release exponent (*n*)** | **Release mechanism** |
| 6 x 3.6 | 0.53 | Anomalous transport |
| 8 x 3.6 | 0.68 | Anomalous transport |
| 10 x 3.6 | 0.98 | Super case II transport |
| Ch 8 x 3.6 | 0.95 | Super case II transport |
| Ch 10 x 3.6 | 1.10 | Super case II transport |
